# Interpretable machine-learning model for Predicting the Convalescent COVID-19 patients with pulmonary diffusing capacity impairment

**DOI:** 10.1186/s12911-023-02192-6

**Published:** 2023-08-29

**Authors:** Fu-qiang Ma, Cong He, Hao-ran Yang, Zuo-wei Hu, He-rong Mao, Cun-yu Fan, Yu Qi, Ji-xian Zhang, Bo Xu

**Affiliations:** 1 Hubei University of Chinese Medicine, Wuhan, 430065 China; 2 Hubei Provincial Hospital of Traditional Chinese Medicine, Wuhan, 430061 China; 3 Affiliated Hospital of Hubei University of Traditional Chinese Medicine, Wuhan, 430061 China; 4 Hubei Province Academy of Traditional Chinese Medicine, Wuhan, 430074 China; 5 School of Software, HuaZhong University of Science and Technology, Wuhan, 430074 China; 6 Wuhan No.1 Hospital, Wuhan, 430022 China; 7 Hubei Provincial Hospital of Integrated Traditional Chinese and Western Medicine, Wuhan, 430015 China

**Keywords:** Interpretable artificial intelligence, Machine learning, COVID-19, Pulmonary diffusing capacity impairment, Maximal voluntary ventilation

## Abstract

**Introduction:**

The COVID-19 patients in the convalescent stage noticeably have pulmonary diffusing capacity impairment (PDCI). The pulmonary diffusing capacity is a frequently-used indicator of the COVID-19 survivors’ prognosis of pulmonary function, but the current studies focusing on prediction of the pulmonary diffusing capacity of these people are limited. The aim of this study was to develop and validate a machine learning (ML) model for predicting PDCI in the COVID-19 patients using routinely available clinical data, thus assisting the clinical diagnosis.

**Methods:**

Collected from a follow-up study from August to September 2021 of 221 hospitalized survivors of COVID-19 18 months after discharge from Wuhan, including the demographic characteristics and clinical examination, the data in this study were randomly separated into a training (80%) data set and a validation (20%) data set. Six popular machine learning models were developed to predict the pulmonary diffusing capacity of patients infected with COVID-19 in the recovery stage. The performance indicators of the model included area under the curve (AUC), Accuracy, Recall, Precision, Positive Predictive Value(PPV), Negative Predictive Value (NPV) and F1. The model with the optimum performance was defined as the optimal model, which was further employed in the interpretability analysis. The MAHAKIL method was utilized to balance the data and optimize the balance of sample distribution, while the RFECV method for feature selection was utilized to select combined features more favorable to machine learning.

**Results:**

A total of 221 COVID-19 survivors were recruited in this study after discharge from hospitals in Wuhan. Of these participants, 117 (52.94%) were female, with a median age of 58.2 years (standard deviation (SD) = 12). After feature selection, 31 of the 37 clinical factors were finally selected for use in constructing the model. Among the six tested ML models, the best performance was accomplished in the XGBoost model, with an AUC of 0.755 and an accuracy of 78.01% after experimental verification. The SHAPELY Additive explanations (SHAP) summary analysis exhibited that hemoglobin (Hb), maximal voluntary ventilation (MVV), severity of illness, platelet (PLT), Uric Acid (UA) and blood urea nitrogen (BUN) were the top six most important factors affecting the XGBoost model decision-making.

**Conclusion:**

The XGBoost model reported here showed a good prognostic prediction ability for PDCI of COVID-19 survivors during the recovery period. Among the interpretation methods based on the importance of SHAP values, Hb and MVV contributed the most to the prediction of PDCI outcomes of COVID-19 survivors in the recovery period.

**Supplementary Information:**

The online version contains supplementary material available at 10.1186/s12911-023-02192-6.

## Introduction

As of November 28, 2022, a novel global pandemic triggered by Corona Virus Disease 2019 (COVID-19) has infected more than 641 million people and claimed 6.63 million lives (https://coronavirus.jhu.edu/). Among the COVID-19 survivors, many have shown disastrous effects on multiple organs and systems [1], but the lung is the organ most susceptible to severe damage from COVID-19 [2]. The convalescent COVID-19 patients have demonstrated particularly pronounced PDCI. Our previous study has found that the incidence of DLCO impairment of the COVID-19 patients reached 57.92% in 18 months after discharge [3]. Studies show that pulmonary diffusing capacity of the COVID-19 patients is also significantly impaired in the 1–24 month recovery phase. Studies also suggest impaired gas-blood exchange in patients discharged after admission for COVID-19 [1, 4–7], and low DLCO may be the result of interstitial or pulmonary vascular abnormalities caused by COVID-19 [8–11]. Therefore, there is an urgent need for a prognostic assessment and early warning system for COVID-19, especially for a model to predict PDCI of the convalescent patients. To solve this problem, establishment of an early warning model to estimate the DLCO of patients is probably an alternative. The current prediction models for COVID-19 are mainly utilized to identify the high-risk groups of the general population [12], diagnose COVID-19 patients [13], and predict the progression of disease severity and mortality [14, 15]. However, the prediction models for PDCI of COVID-19 patients are still in deficiency.

Machine learning analysis is based on various data mining algorithms of different types and formats to characterize the data features in a more scientific way and gain better insight into data trends and recognized values [16]. At present, machine learning has been widely used in multiple domains of life and production in human society, including the analysis and prediction of energy consumption by sewage treatment and the prediction of building materials and composite properties [17–20]. Currently, ML is also widely used in healthcare data analysis [21]. However, the “black box” problem makes it difficult to integrate AI and AI technology with clinical practice. The “black box” of medicine allows no clinicians to review the quality of training labels or data, which is contrary to the rules followed by evidence-based medicine. Therefore, the capability of correctly interpreting the output of a predictive model is extremely important to generate appropriate user trust, provide insights on how to improve the model, and support an understanding of the modeling process so that AI can be combined with human intelligence to fully exploit the potential and productivity advantages of AI.

In interpretability machine learning, SHAP is ascribed to the post-interpreting method of model, and its central ideology is to calculate the marginal contribution of features to the output of the model, and then elucidate the “black box model” at both global and local levels. The interpretability of SHAP is essential to enhance the trust of healthcare professionals, because it exhibits sufficient reason to make predictions and how parameters contribute to the model [22]. The interpretation method based on the importance of SHAP value features can help medical researchers understand the decision-making criteria of ML models. The ability of making use of large data sets and predictive models enables clinicians to diagnose, treat and predict their patients in a more confident manner [23].

However, most ML studies worked hard to improve performance by increasing the model complexity, leading to uncertainties in the way how ML operates and makes decisions [24–26]. To improve interpretability of the ML models, this study adopted the most popular feature importance estimation in the explainabilty researches [27, 28]. We tried to rank the features according to their importance and used the TreeSHAP method proposed by Lundberg et al. to analyze the clinical features [29].

Therefore, the aim of this investigation is to develop and validate an interpretable ML model based on clinical variables to evaluate the risk of PDCI of the COVID-19 patients in recovery. This study had been reviewed and approved by the Ethics Committee of Hubei Provincial Hospital of Integrated Traditional Chinese and Western Medicine (2,020,009). All participants provided their written or verbal informed consents prior to the study.

## Methods

### Study design and data set

From August to September 2021, a follow-up study had been conducted on the COVID-19 survivors 18 months after discharge from Hubei Provincial Hospital of Integrated Traditional Chinese and Western Medicine. A total of 221 survivors were contacted according to their time of discharge. Related clinical data of survivors, including demographic characteristics (age, sex and body mass index (BMI)) and clinical examination indicators (lung function, chest HRCT, antibody titers and various biochemical indicators), were collected by the well-trained physicians.

**Fig. 1:**
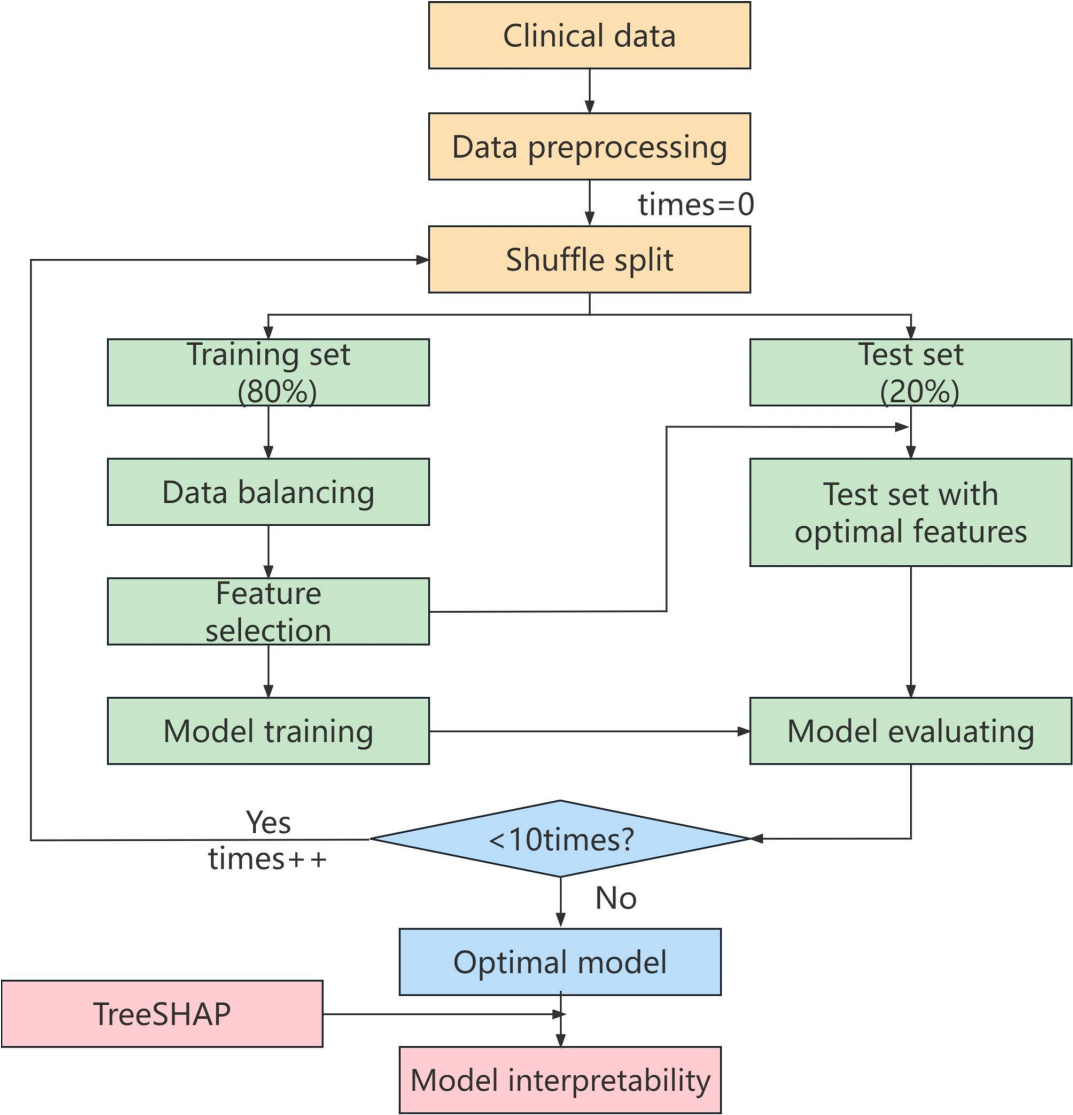
Flow diagram of model design

The three-step primary procedures were performed in this investigation. Firstly, six popular machine learning models were used to predict pulmonary diffusing capacity of patients at convalescent stage of COVID-19. Secondly, the performance of the six ML models was tested with a selection of indicators including AUC, Accuracy, Recall, Precision, PPV, NPV and F1, and then the model with the optimum performance was defined as the optimal model. Finally, we used the MAHAKIL method for data balance processing to optimize the balance of sample distribution, while the RFECV method for feature selection, which could choose combined features conducive to machine learning. The overall workflow of this study is shown in Fig. 1.

### Patients and outcomes

The criteria for inclusion of survivors in this study were determined in accordance with the COVID-19 management protocols of the World Health Organization (WHO) and National Health Commission of the People’s Republic of China [30, 31](http://en.nhc.gov.cn/2020-03/29/c_78469.htm).

The severity of COVID-19 is measured as follows:

1. Mild cases: without symptoms and signs of severe and critical infection;
2. Moderate cases: with fever, respiratory symptoms and suggested.

Pneumonia via chest radiology;

3. Severe cases:

- Breathing difficulties, respiratory rate ≥ 30 bpm;
- SpO2 ≤ 93% at rest;
- PaO2/FiO2 ratio ≤ 300 mmHg.

4. Critical cases:

- Respiratory failure requiring mechanical ventilation;
- Shock;
- Multi-organ failure requiring intensive care.

The primary endpoint of this study was the area under the receiver operating characteristic curve (AUROC) of the model’s prediction, while the secondary endpoints were Accuracy, Recall, Precision, PPV, NPV and F1 score of the model’s prediction.

### Data collection

Clinical data on the COVID-19 survivors include demographics, medical history, laboratory tests, and scoring system and outcomes of illness severity. Demographic characteristics were extracted covering gender, age, height and body weight. Then, data were collected on comorbidities, including heart failure, anemia and chronic obstructive pulmonary disease (COPD). The laboratory tests abstracted include white blood cells (WBC), Hb, PLT, N%, L%, LY#, IgM, IgG, proBNP, alanine transaminase (ALT), aspartate aminotransferase (AST), Alb, BUN, Cr, UA, HbA1c, Normal unilateral & bilateral score, forced vital capacity (FVC), forced expiratory volume in one second (FEV1), FEV1/FVC, MVV, DLCO, tubercle, ground-glass opacity (GGO), fibrosis, etc. The severity of illness is scaled from 1 to 4.

### Feature selection and data preprocessing

High dimensional data analysis modeling poses a challenge for the researchers in the field of data mining. Feature selection technology provides an effective solution this problem by removing irrelevant and redundant data, which renders it possible to reduce the computation time, improve the learning accuracy and better understand the learning model [22]. Cai et al. compared and analyzed some state-of-art feature selection methods on two high-dimensional gene-expression data sets through experiments [27], which found that recursive feature elimination (RFE) could achieve higher accuracy than other feature selection methods. In this regard, we chose RFECV, a Cross Validation version of RFE. The purpose of adding Cross Validation is to select the best number of features, which often requires manual trial and error to obtain the best number of features in studies using RFE. In our study, the RFECV method was used to cyclically remove medical features that were detrimental to the ability of the model in learning to predict the pulmonary diffusing capacity until the assembled features enabled the model to perform optimally. Followed by feature selection, a total of 31 among the 37 clinical factors were ultimately selected for model construction.

### Model development and validation

Randomly, the data were separated into a training data set (80%) and a validation data set (20%). Firstly, up-sampling by the MAHAKIL method was utilized to balance the number of samples of different classes in the training set. Secondly, the RFECV method was utilized to choose the optimized combination of features. Then, the selected features from the balanced training set were input into the machine learning model for training and modeling, and the grid-search method was utilized to ensure the validity of the combination of parameters during training. Finally, the trained ML model was utilized to predict and evaluate the data results of the test set, where the features were also processed as the optimal combination of features. In addition, we integrated the overall data, ranked the importance of features by taking XGBoost as the base model and using the TreeExplainer method, and combined with the calculating principle of the SHAP interaction values to further explain the reasons why these features were considered significant.

**Fig. 2:**
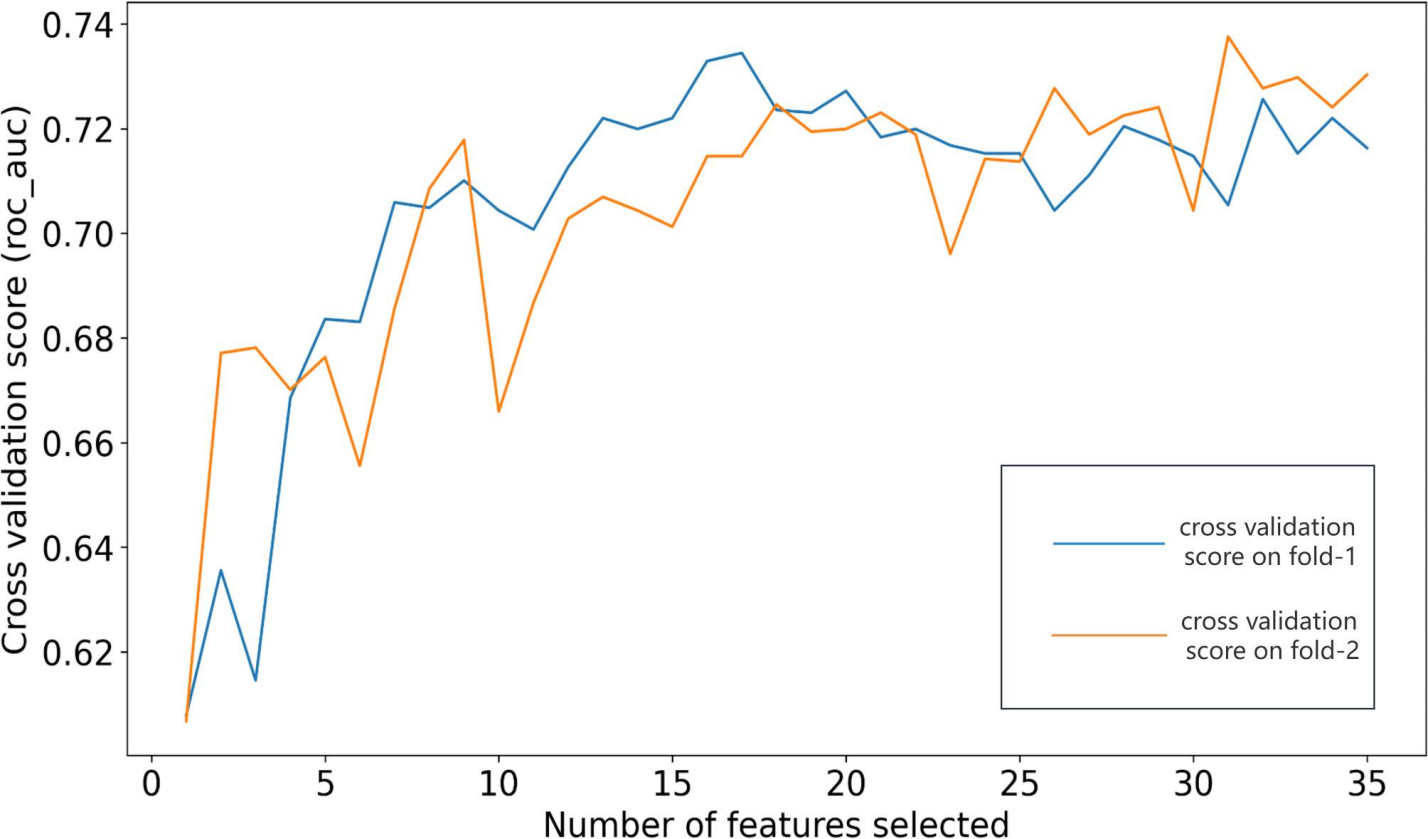
Change of ROC_AUC of the XGBoost model and number of features

The XGBoost model ROC_AUC changes corresponding to the number of features are shown in Fig. 2. After feature selection via the RFECV method, 31 were selected as the optimal combined features.

### Model interpretability

It’s extremely significant to open the black box of ML to improve the compliance and transparency of the ML decision-making process for healthcare workers [32]. Therefore, we took XGBoost with the best performance in AUC evaluation index as the base model, the optimal combined features after feature selection and labels as the input, and use the TreeExplainer method to sort the SHAP values of features. The SHAP value summary diagram of medical characteristics is shown in Fig. 4.

### Statistical analysis

The count data were described with the number of cases (%), and Pearson chi-square test was utilized for inter-group comparison. Measurement data accorded with the normal distribution were expressed as the median and interquartile range (M (P25, P75)) by t-test or ANOVA, while the Mann-Whitney U test was used between groups. After feature selection and data preprocessing, six popular machine learning models were developed to predict PDCI of survivors recovering from COVID-19. Overall, performance of each model was assessed by AC, Accuracy, Precision, Recall, PPV, NPV and F1 measurements. Ultimately, the model was explained by the TreeExplainer method.

SPSS 25.0 (IBM, Armonk, New York, USA) was applied for statistical analysis. All statistical tests were two-sided, and *P* < 0.05 was considered statistically significant.

## Results

### Clinical characteristics

A total of 221 COVID-19 survivors were included in this study (Mild cases, n = 93; Moderate cases, n = 58; Severe cases, n = 54; Critical cases, n = 16). The median age of the subjects was 58.2 [standard deviation (SD) = 12]. Among them, 104 survivors (47.06%) were male and 117 (52.94%) were female, with an average BMI of 24.62[standard deviation (SD) = 3.5]. The incidence of PDCI in COVID-19 survivors was 57.92% as shown in Table 1 (from J Infect. 2022 Feb; 84(2):e16-e18 Table 1 PMID: 34,963,637).

**Table 1:** Clinical characteristics of survivors with impaired and normal DLCO

	All patients M (P25,P75)	Patients with impaired DLCO	Patients with normal DLCO	t or x^2^	*P* value
Age	61(51,66)	62(51,67)	59(47.5,66.0)	-0.979	0.328
Gender					
Male (%)	104	42	62	24.114	0.000
Female (%)	117	86	31		
Illness severity					
mild	93	43	50	19.476	0.000
moderate	58	31	27		
severe	54	39	15		
critical	16	15	1		
WBC	5.640(4.810,6.530)	5.650(4.923,6.500)	5.695(4.810,6.450)	-0.366	0.714
Hb	135.000(127.000,145.000)	131.00(123.250,139.750)	139(132,151)	-5.200	0.000
PLT	184.000(158.000,219.000)	198.500(159.250,229.750)	179(156,209.750)	-2.545	0.011
N%	56.140(50.540,61.300)	56.600(52.685,62.075)	55.550(50.425,60.300)	-1.556	0.120
L%	32.4 ± 6.49	32.290 ± 6.415	32.618 ± 6.643	0.369	0.712
LY#	1.790(1.500,2.120)	1.790(1.493,2.063)	1.785(1.500,2.173)	-0.035	0.972
IgM	0.540(0.220,1.470)	0.500(0.220,1.518)	0.515(0.233,1.148)	-0.142	0.887
IgG	138.340(67.640,210.930)	146.100( 73.298,217.485)	135.125(58.258,207.860)	-0.859	0.390
proBNP	105.300(81.250,169.200)	106.400(81.250,172.200)	103.340(80.633,166.075)	-0.213	0.831
ALT	13.000(10.000,21.000)	12.000(9.000,20.000)	13.000(10.000,21.000)	-1.774	0.081
AST	21.000(17.000,25.000)	20.500(17.00,25.750)	22.000(17.000,25.000)	-1.166	0.868
Alb	44.600(43.300,46.000)	44.500(43.425,45.875)	44.900(43.425,46.375)	-1.282	0.200
BUN	5.430(4.600,6.340)	5.525(4.760,6.345)	5.275(4.228,6.525)	-1.218	0.223
Cr	66.800(56.400,80.000)	63.950(54.100,78.650)	71.050(59.400,82.475)	-2.611	0.009
UA	343.7(289.600,409.000)	339.450(287.075,400.475)	368.450(289.625,416.800)	-1.697	0.090
HbA1c	5.500(5.200,5.900)	5.600(5.300,6.000)	5.400(5.100,5.900)	-2.199	0.028
FVC	100.28 ± 16.33	98.055 ± 16.615	103.520 ± 15.468	2.476	0.014
FEV1	101.47 ± 17.62	99.495 ± 18.569	104.361 ± 18.928	2.033	0.043
FEV1/FVC	83.800(79.000,89.320)	84.500(79.300,89.468)	82.650(78.100,89.315)	-0.971	0.332
MVV	93.52 ± 24.8	88.013 ± 25.142	101.278 ± 22.311	4.032	0.000
BMI kg•m^− 2^	24.78(22.35,26.81)	24.140(21.915,26.583)	25.775(22.965,27.033)	-2.078	0.038
CT SCORE	2(1, 3)	2.000(1.000,4.000)	2.000(0.000,3.000)	-2.439	0.015

### Model development and validation

After feature selection, we utilized 31 alternative factors for model construction, and among the six ML models tested by the team. Compared with GBDT (A.C. 0.67), KNN (A.C. 0.63), RandomForest (A.C. 0.70) and SVC (A.C. 0.70), MLP (A.C. 0.69), XGBoost (A.C. 0.75) has better DLCO predicting ability for COVID-19 survivors. Table 2 exhibits that XGBoost performs optimally in AUC, Accuracy, Recall, Precision, PPV, NPV and F1. After experimental verification, the model has an AUC of 0.755 and an Accuracy of 78.01%. The SHAP summary analysis demonstrated that Hb, MVV, severity of illness, PLT, UA and BUN were the top six key factors affecting the decision-making of XGBoost model.

**Table 2:** Experimental results of different classifiers

Classifier	AUC	Accuracy	Recall	Precision	PPV	NPV	F1
GBDT	0.6787	0.7044	0.6787	0.6938	0.6429	0.5484	0.6764
KNN	0.6392	0.6487	0.6392	0.6378	0.5333	0.7000	0.6309
RandomForest	0.7011	0.7376	0.7011	0.7434	0.9000	0.6571	0.7009
SVC	0.7085	0.7358	0.7085	0.7224	0.6522	0.7273	0.7104
MLP	0.6912	0.7066	0.6912	0.6937	0.6923	0.7500	0.6872
XGBoost	**0.7550**	**0.7801**	**0.7550**	**0.7755**	**0.7083**	**0.8095**	**0.7572**

**Fig. 3:**
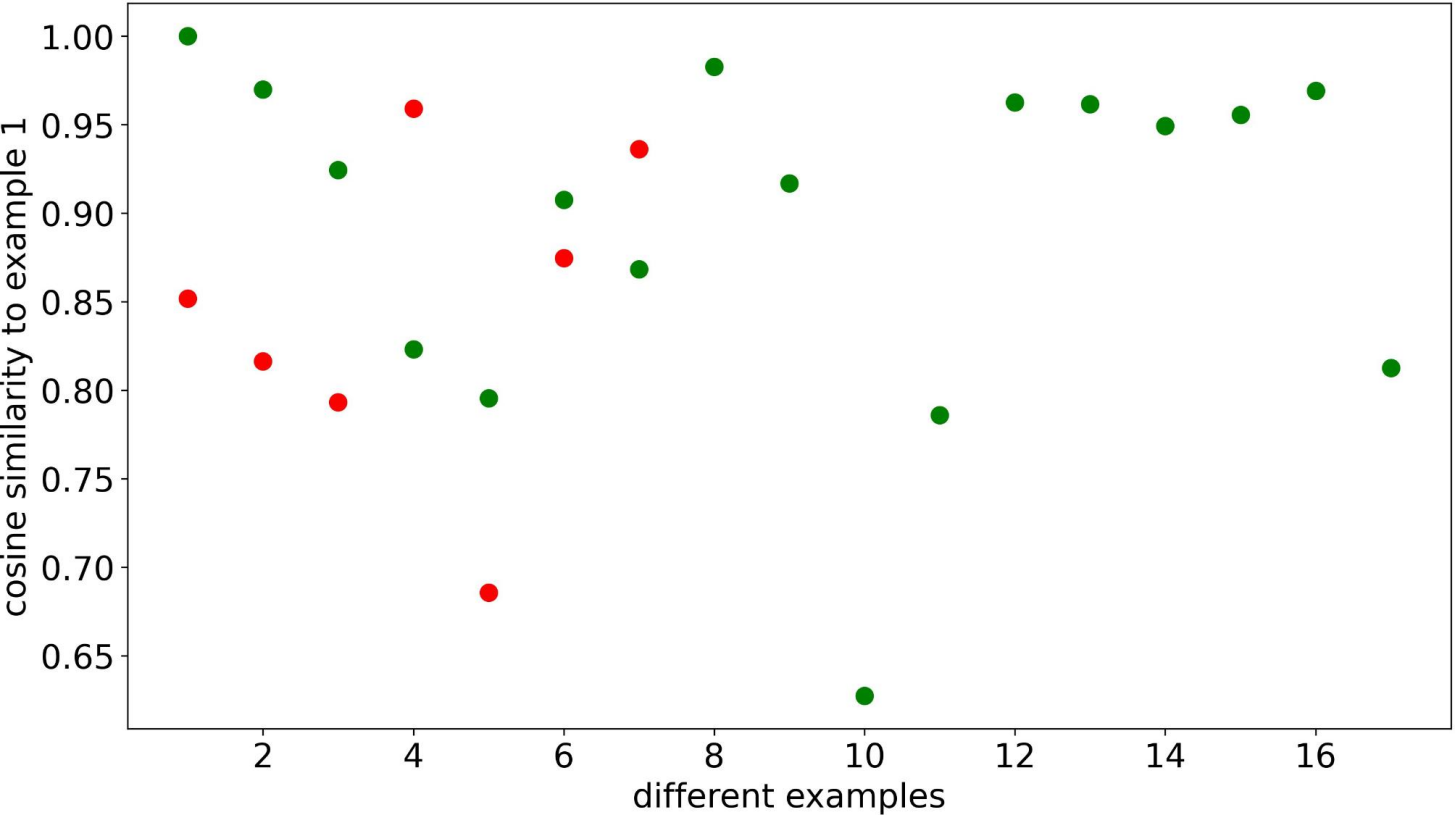
Cosine similarity of XGBoost model

The cosine similarity of XGBoost model is shown in Fig. 3. The green dots are samples that are correctly believed to be 0 (0 represents healthy people), and the red dots are samples that are incorrectly believed to be 0 (patients diagnosed as healthy people). It’s found that the reason for the misjudgment is that the misjudged sample features are quite similar to the true 0 sample features (the vast majority are greater than 0.8). That is to say, one of the important reasons for misjudgment is that these patients’ symptoms or some of the detected indicators in the body have a very high degree of similarity with normal people.

### Model interpretability

**Fig. 4:**
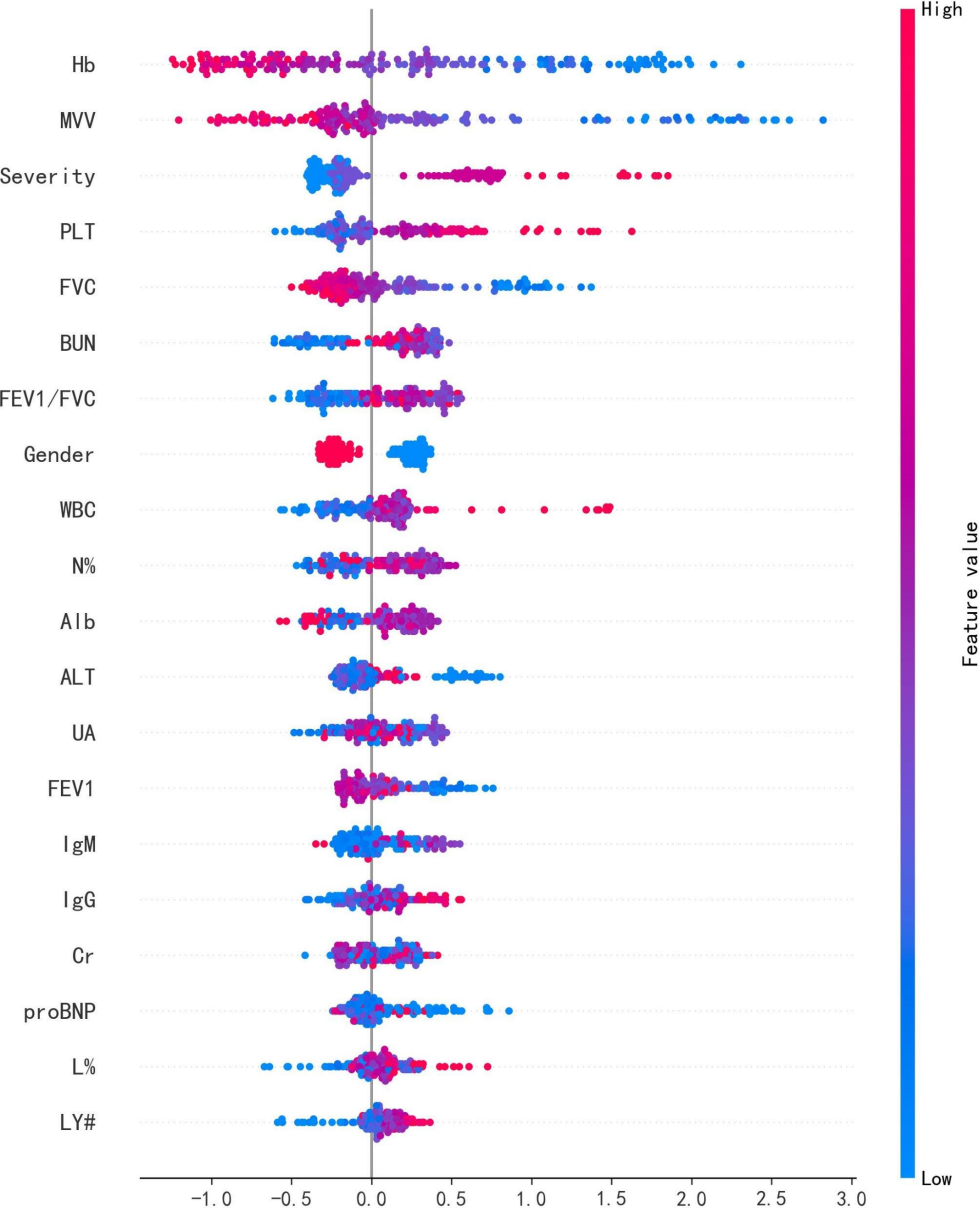
SHAP value summary diagram of medical characteristics

**Fig. 5:**
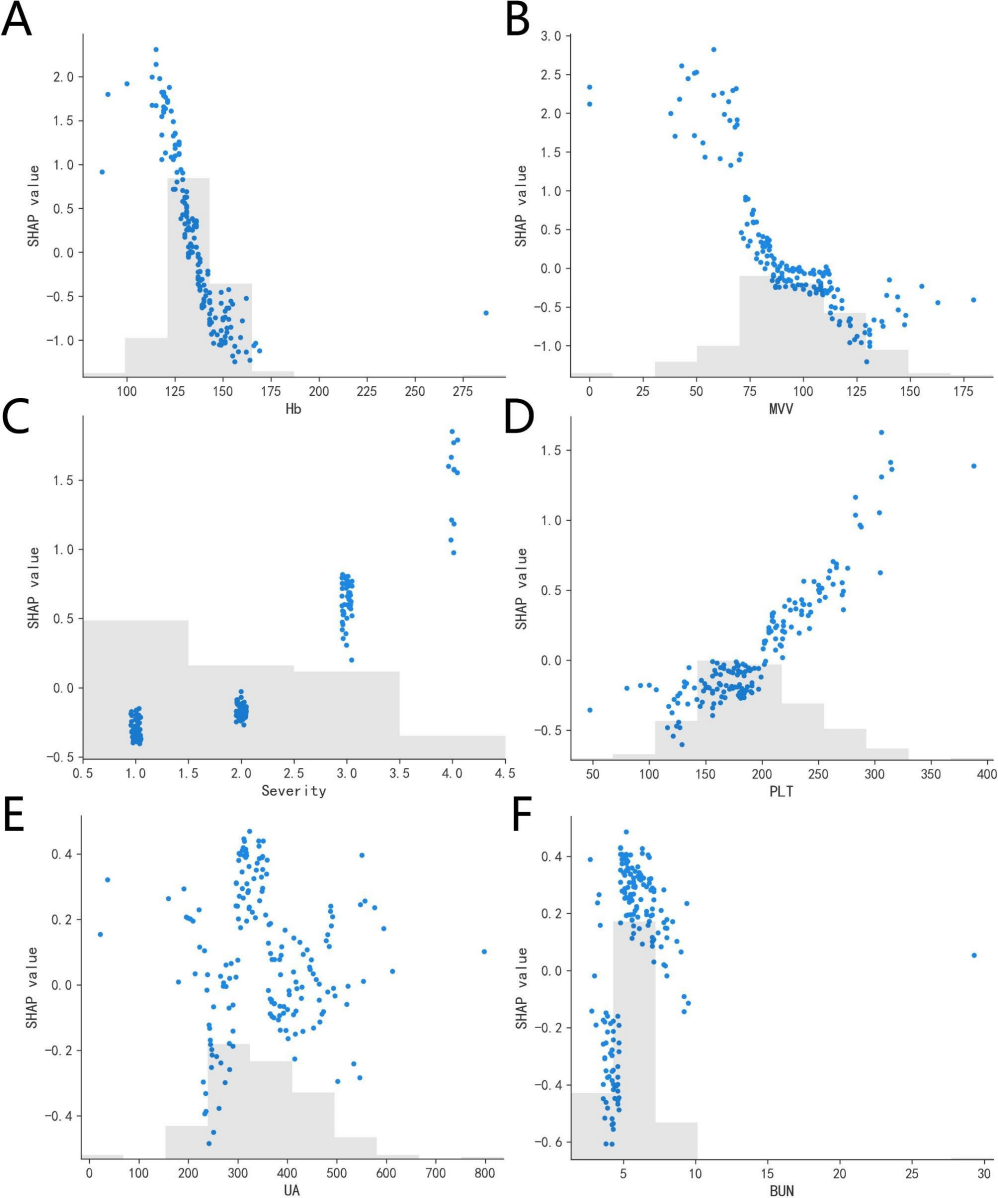
Top 6 clinical features in SHAP values of XGBoost. **A** Hb, **B** MVV, **C** severity of illness, **D** PLT, **E** UA, **F** BUN. Values are plotted with a scatter plot. The morbidity of PDCI predicted by the model will be increased when the SHAP value of the feature is > 0, and the disease-free rate of PDCI predicted by the model will be increased when the SHAP value is < 0. *MVV*: maximal voluntary ventilation; *HB*: hemoglobin, *PLT*: platelets; *BUN*: blood urea nitrogen; *UA*: uric acid

Figure 4 shows the SHAP summary diagram, which ranks the factors according to their importance to the predicted incidence of the validation cohort. The SHAP summary analysis revealed that Hb, MVV, level, PLT, UA and BUN were the top six most pivotal factors affecting the XGBoost model decision. Figure 5 also shows the correlation between the six factors and the prediction of PDCI occurred in the COVID-19 survivors. The SHAP values above zero for these six characteristics indicate an increased risk of PDCI. Hb and MVV were negatively correlated with DLCO, while severity of illness, PLT, UA and BUN were positively correlated.

The SHAP values in the validation set were utilized to evaluate the feature importance of the XGBoost classifier. Each dot represents 1 patient and is accumulated vertically to describe density. Colors represent high and low values for each element, with dark colors representing higher values and light colors lower values. The X-axis of the diagram represents the SHAP value. A positive SHAP value indicates a positive contribution to the prediction model and a high probability of PDCI occurrence, and vice versa.

**Fig. 6:**
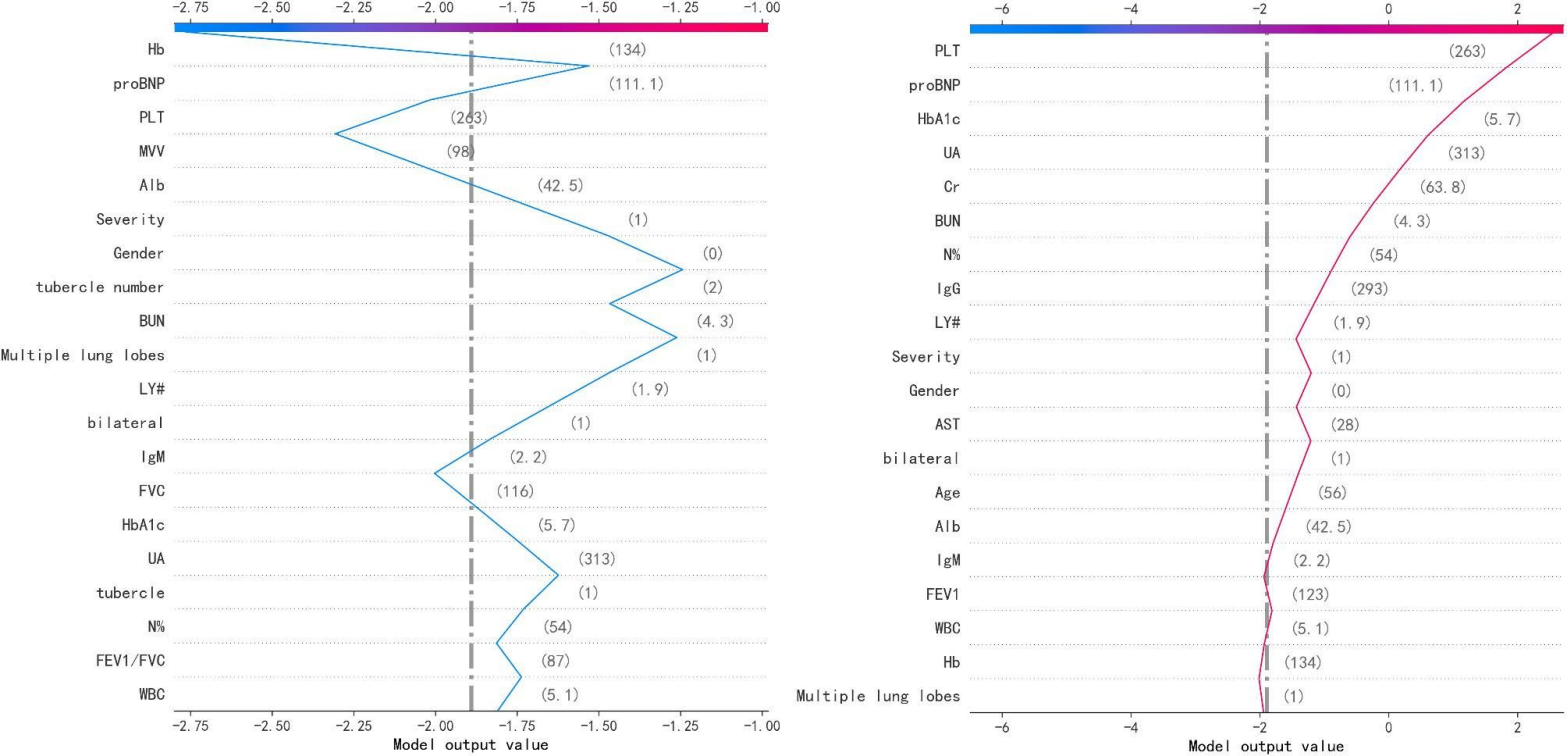
Output decision chain of the XGBoost model

Finally, we plotted the XGBoost decision-making process against the SHAP values, as shown in Fig. 6. The gray vertical line in the middle of the decision graph marks the model’s base value, and the colored line is for prediction, indicating whether each feature moves the output value to a value higher or lower than the average prediction. The eigenvalues next to the prediction line can be taken as the reference. Starting at the bottom of the graph, the prediction line shows how the SHAP value grows from the base value to the model final score at the top of the graph. The blue broken line is the decision process of predicting a normal object, and the red broken line is the decision process of predicting an exception object.

## Discussion

The aim of this ML-based modeling study was to develop a valid, stable and interpretable model for predicting the incidence of PDCI in COVID-19 survivors in the recovery phase. The findings of the study manifested that the XGBoost model was the most reliable and accurate among all the tested models, with an AUC of 0.755 and an Accuracy of 78.01%. We also found that Hb, MVV, severity of illness, PLT, UA and BUN were the top six key factors influencing the XGBoost model decision-making. Overall, our study demonstrated that it is possible to predict the PDCI incidence of COVID-19 survivors using routinely collected clinical data.

As the global pandemic of COVID-19 continues to exert damaging effects on our world and the number of patients recovering from the disease increases, studies have found that shortness of breath and dyspnea are the most common sequelae among those who have survived hospitalization with COVID-19 due to the presence of PDCI [7]. Therefore, DLCO-based pulmonary function testing can be regarded as a useful parameter to differentiate those at risk of pulmonary sequelae [33]. However, previous studies on COVID-19 mainly focus on the analysis of risk factors and the prediction of mortality of mild-to-moderate cases [34, 35], without a prediction model for PDCI in COVID-19 survivors. Consequently, it’s essential to develop and validate the risk-level outcome prediction models to evaluate the pulmonary function status of COVID-19 survivors.

Apart from using a machine learning model to predict the pulmonary diffusing capacity of patients recovering from COVID-19, our investigation further applied the model to interpretability analysis. Because the internal logic and operating mechanisms of ML models are concealed from users, this uncertainty poses challenges for healthcare workers in applying the machine learning systems in reality. In this study, we used the interpretation method based on the importance of the SHAP value features to help medical researchers understand the decision-making criteria of ML models [36–38], enhance the credibility of medical professionals in ML, and coordinate the contradictions or inconsistencies between the knowledge structural elements of machines and human beings with prior knowledge. We adopted the TreeSHAP method [6], which is an effective evaluative method for the importance of tree model features based on the SHAPELY value of classical game theory. The SHAP summary analysis showed the six most important factors of the XGBoost model. Among them, MVV is the most important indicator of lung reserve function, which is closely related to activity endurance. The most serious sequelae of the COVID-19 patients are shortness of breath and dyspnea in the wake of activities, and the significantly decreased pulmonary function reserve [39]. This study confirmed that MVV was positively correlated with the pulmonary diffusing function of COVID-19 survivors. The MVV value of COVID-19 patients with normal diffusing function was significantly higher than that of patients with impaired pulmonary diffusing capacity, which reveals the importance of strengthening pulmonary rehabilitation exercises and increasing pulmonary function reserve in COVID-19 patients during rehabilitation. Studies have found that Hb, a parameter closely related to organ perfusion, alveolar ventilation and blood flow ratio, has greatly contributed to the prediction of pulmonary function outcomes in patients with COVID-19 after recovery. In this study, after the correction of Hb, Hb of COVID-19 patients with decreased pulmonary diffusing function was normal low value or anemia, indicating that there is a long-term imbalance of pulmonary perfusion and ventilation ratio in COVID-19 patients, to which due attention should be paid.

In addition, PLT and severity of illness were negatively correlated with pulmonary diffusing function. The more severe the disease was, the higher the normal value of PLT was, and vice versa. Studies have confirmed that PLT activation is involved in the formation of inflammatory microvascular thrombosis of patients with COVID-19 and is closely related to respiratory failure in COVID-19 patients [10, 40–44]. However, one and a half years later, our study found that PLT was still closely related to PDCI of COVID-19 survivors. Consistent with our previous study [45], these observations suggest that clinically obtained MVV, PLT, Hb and severity of illness are the key factors for using the XGBoost model to predict pulmonary function status in COVID-19 survivors. Besides, compared with the indicators directly affecting pulmonary function, the SHAP pooled analysis exhibited that the increased UA and BUN may be correlated with a growing risk of the retrogressive pulmonary diffusing capacity of the COVID-19 patients.

Currently, the combined use of high-frequency biological data streams and artificial intelligence (AI) indicates a promising application for predicting the diffusing capacity of lungs, which makes it possible for early identification of pulmonary capacity recovery of COVID-19 patients [46–48]. However, this study is still subjected to some limitations. First of all, no causal connection between variables and the pulmonary diffusing capacity can be drawn based on the modeling and retrospective design in this study. Secondly, the predictive efficiency of the current models may work differently if the racial and ethnic characteristics of the subjects are not identical in the study. Moreover, it is difficult to obtain more relevant data due to the privacy of COVID-19 patients, leading to a lack of proper external validation of our prediction model, which will affect the credibility of the XGBoost model. Finally, although the findings showed that the model had learned the medical rules in the data, the data expansion is an urgent need in the future to improve the model’s performance.

## Conclusion

This article analyzes the pulmonary capacity and other clinical indicators of COVID-19 survivors. Six popular machine learning models were utilized to predict pulmonary diffusing capacity of COVID-19 patients at recovery stage, among which the XGBoost model showed favorable predictive ability. It adopts an optimized second-order Taylor expansion, which can better fit complex nonlinear data sets by using second-order functions. In addition, XGBoost explicitly adds regular terms to the objective function to reduce variance and prevent overfitting of the model. In interpretable machine learning, Hb, and MVV contributed most to the prediction of PDCI outcomes of COVID-19 survivors in the convalescence period. The interpretation methods based on the importance of SHAP values can help doctors better familiarize with the basic concepts and indicators of machine learning and their potential applications in clinical practices, and then readily accept the growing integration of AI and machine learning with modern medicine.

### Electronic supplementary material

Below is the link to the electronic supplementary material.

Supplementary Material 1

## Data Availability

The datasets used and analyzed during the current study available from the corresponding author on reasonable request.
