# Supplementary material for: Interpretable machine-learning model for Predicting the Convalescent COVID-19 patients with pulmonary diffusing capacity impairment
Source: BMC Med Inform Decis Mak. 2023 Aug 29;23:169. doi: 10.1186/s12911-023-02192-6 (PMC10466769; doi:10.1186/s12911-023-02192-6)
Supplement: Supplementary file 1 — Supplementary Material 1 [file 12911_2023_2192_MOESM1_ESM.pdf]

Experimental results and parameters of different classifiers

classifier	data source	random_seed	auc	accuracy	recall	precision	f1	
GBDT	preprocessed_data	200	0.690	0.733	0.690	0.725	0.697	n_estimators=1155,<br>max_depth=7,<br>min_samples_split=2,<br>min_samples_leaf=2,<br>max_features=6,<br>subsample=0.55
		201	0.675	0.673	0.675	0.680	0.672	
		202	0.743	0.760	0.743	0.734	0.737	
		203	0.667	0.722	0.667	0.699	0.674	
		204	0.709	0.747	0.709	0.680	0.688	
		205	0.732	0.733	0.732	0.739	0.731	
		206	0.656	0.651	0.656	0.658	0.650	
		207	0.609	0.627	0.609	0.622	0.605	
		208	0.621	0.649	0.621	0.620	0.620	
		209	0.685	0.749	0.685	0.781	0.690	
		**average**	**0.6787**	**0.7044**	**0.6787**	**0.6938**	**0.6764**	
SVC	normalized_data	200	0.741	0.778	0.741	0.775	0.750	C= 100,<br>gamma=0.01
		201	0.690	0.689	0.690	0.690	0.689	
		202	0.733	0.756	0.733	0.726	0.729	
		203	0.664	0.711	0.664	0.683	0.669	
		204	0.714	0.800	0.714	0.729	0.720	
		205	0.752	0.756	0.752	0.783	0.748	
		206	0.667	0.667	0.667	0.666	0.666	
		207	0.770	0.778	0.770	0.778	0.772	
		208	0.643	0.667	0.643	0.640	0.641	
		209	0.711	0.756	0.711	0.754	0.720	
		**average**	**0.7085**	**0.7358**	**0.7085**	**0.7224**	**0.7104**	
KNN	normalized_data	200	0.664	0.711	0.664	0.697	0.669	weights='distance',<br>n_neighbors=3,<br>p=11
		201	0.602	0.600	0.602	0.604	0.598	
		202	0.667	0.667	0.667	0.650	0.649	
		203	0.540	0.533	0.540	0.537	0.525	
		204	0.761	0.778	0.761	0.717	0.730	
		205	0.641	0.644	0.641	0.654	0.636	
		206	0.643	0.644	0.643	0.643	0.643	
		207	0.655	0.644	0.655	0.657	0.644	
		208	0.609	0.622	0.609	0.602	0.603	
		209	0.610	0.644	0.610	0.617	0.612	
		**average**	**0.6392**	**0.6487**	**0.6392**	**0.6378**	**0.6309**	
RandomForest	preprocessed_data	200	0.738	0.778	0.738	0.779	0.748	bootstrap= True ,<br>max_depth= 22,<br>max_features= 'auto',<br>min_samples_leaf= 13,<br>min_samples_split= 3,<br>n_estimators= 400
		201	0.721	0.716	0.721	0.774	0.702	
		202	0.760	0.791	0.760	0.766	0.763	
		203	0.650	0.724	0.650	0.717	0.657	
		204	0.756	0.822	0.756	0.762	0.758	
		205	0.704	0.709	0.704	0.756	0.692	
		206	0.710	0.713	0.710	0.714	0.710	
		207	0.634	0.656	0.634	0.663	0.628	
		208	0.648	0.707	0.648	0.680	0.654	
		209	0.690	0.760	0.690	0.823	0.697	
		**average**	**0.7011**	**0.7376**	**0.7011**	**0.7434**	**0.7009**	
mlp	normalized_data	200	0.693	0.733	0.693	0.721	0.700	solver='adam',<br>alpha=0.001,<br>hidden_layer_sizes=(10, 30),<br>random_state=1,<br>activation='relu',<br>batch_size='auto',<br>beta_1=0.9,learning_rate='constant',<br>learning_rate_init=0.001,<br>max_iter=200,<br>momentum=0.9,
		201	0.690	0.689	0.690	0.690	0.689	
		202	0.667	0.689	0.667	0.658	0.661	
		203	0.581	0.622	0.581	0.583	0.582	
		204	0.790	0.822	0.790	0.761	0.773	
		205	0.708	0.711	0.708	0.733	0.702	
		206	0.714	0.711	0.714	0.714	0.711	
		207	0.680	0.689	0.680	0.685	0.681	
		208	0.637	0.622	0.637	0.625	0.615	
		209	0.752	0.778	0.752	0.767	0.758	
		**average**	**0.6912**	**0.7066**	**0.6912**	**0.6937**	**0.6872**	
XGBoost	preprocessed_data	200	0.776	0.822	0.776	0.852	0.793	max_depth=5,<br>learning_rate=0.1,<br>n_estimators=800,<br>objective='binary:logistic',<br>nthread=-1,<br>gamma=0,<br>min_child_weight=0,<br>max_delta_step=0.5,<br>subsample=0.2,<br>colsample_bytree=1,
		201	0.760	0.756	0.760	0.803	0.748	
		202	0.717	0.756	0.717	0.725	0.720	
		203	0.712	0.756	0.712	0.737	0.720	
		204	0.774	0.844	0.774	0.793	0.783	
		205	0.776	0.778	0.776	0.787	0.775	
		206	0.759	0.756	0.759	0.759	0.756	
		207	0.725	0.733	0.725	0.731	0.727	
		208	0.681	0.733	0.681	0.712	0.689	
		209	0.870	0.867	0.870	0.856	0.861	
		**average**	**0.755**	**0.7801**	**0.755**	**0.7755**	**0.7572**	
